# Reclassification of VUS in *BRCA1* and *BRCA2* using the new *BRCA1*/*BRCA2* ENIGMA track set demonstrates the superiority of ClinGen ENIGMA Expert Panel specifications over the standard ACMG/AMP classification system

**DOI:** 10.1016/j.gimo.2024.101961

**Published:** 2025-01-07

**Authors:** Anna Benet-Pagès, Andreas Laner, Luis R. Nassar, Tobias Wohlfrom, Verena Steinke-Lange, Maximilian Haeussler, Elke Holinski-Feder

**Affiliations:** 1 Medical Genetics Center (MGZ), Munich, Germany; 2 Institute of Neurogenomics, Helmholtz Zentrum München, German Research Centre for Environmental Health, Munich, Germany; 3 Genomics Institute, University of California Santa Cruz, Santa Cruz, CA; 4 Department of Medicine IV, Klinikum der Universität, Ludwig-Maximilians University, Munich, Germany

**Keywords:** BRCA1, BRCA2, ENIGMA, UCSC Genome Browser, VUS

## Abstract

**Purpose:**

Variants of uncertain significance (VUS) are considered one of the most significant impediments to the translation of genetic test results into precise clinical recommendations. The 2015 American College of Medical Genetics and Genomics/Association for Molecular Pathology (ACMG/AMP) classification guidelines established a general framework for the assessment and classification of genetic variants; yet, gene-specific specifications are needed to enable better variant classification to reduce the number of VUS. The process of gene-specific adaptations of the ACMG/AMP codes is led and accompanied by ClinGen and implemented by Variant Curation Expert Panels (VCEP). The Evidence-based Network for the Interpretation of Germline Mutant Alleles (ENIGMA) VCEP recently published its specifications for the *BRCA1* (HGNC:1100) and *BRCA2* (HGNC:1101) genes. We investigated the differences in reclassification between the ENIGMA specifications and the standard ACMG/AMP classification system in a clinical setting.

**Methods:**

We reclassified 121 VUS identified in these genes with the latest annotation data using the standard ACMG/AMP classification system and recommendations from the Sequence Variant Interpretation and the ENIGMA specifications. To simplify the reevaluation process, we have created a University of California Santa Cruz Genome Browser track hub that displays the exact data points required for variant classification using the ENIGMA VCEP specifications at the exon and variant level (https://genome.ucsc.edu/s/abenet/BRCA.ENIGMA.hg19).

**Results:**

By comparing the codes used and their different weighting in the 2 approaches, we were able to demonstrate the superiority of the application of ENIGMA VCEP specifications, which resulted in a dramatic reduction of VUS (83.5% ENIGMA VCEP vs 20% ACMG/AMP + Sequence Variant Interpretation).

**Conclusion:**

For the diagnostic analysis of the *BRCA1* and *BRCA2* genes, the use of the ENIGMA VCEP specifications gives the best possible result in the clinical translation of genetic variants. The University of California Santa Cruz Genome Browser *BRCA1*/*BRCA2* ENIGMA track set significantly simplified the interpretation process.

## Introduction

Genome and exome sequencing have yielded an extensive number of DNA variants in the human population.^1^^,^^2^ Of the approximately 4 million missense variants discovered so far, only about 2% have a clear clinical classification as benign (B) or pathogenic (P); with the majority of observed missense variants having no clear clinical consequences. The scenario is even worse for intronic and intragenic noncoding variants, most of them lacking clinical significance except for a few functionally characterized variants (usually near the exon/intron boundaries) that affect splicing.^3^ These so-called variants of uncertain significance (VUS) pose a major challenge for clinical reporting, namely answering the fundamental question: is the variant pathogenic or not?^4^

Accurate determination of the pathogenicity of a variant detected in a diagnostic test is of great importance because it defines the application of targeted therapies and personalized interventions, particularly in hereditary tumor syndromes.^5^^,^^6^ Worryingly, Welsh et al^7^ reported that cancer-free patients who were diagnosed with VUS as part of a *BRCA1* and *BRCA2* diagnostic test were more likely to be treated surgically than patients without VUS, although many VUS were later found to be benign. Although a more recent meta-study indicated that the number of individuals with clinically inadequate treatment was not significant,^8^ the American College of Medical Genetics and Genomics/Association for Molecular Pathology (ACMG/AMP)^9^ and the National Comprehensive Cancer Network^10^ guidelines recommend that VUS results should never be used to guide medical decisions.

In a landmark publication in 2015, Richards et al^9^ provided guidelines for the interpretation of sequence variants by establishing a detailed framework for variant classification applicable to a broad number of genes, inheritance patterns, and diseases (ACMG/AMP classification system). Subsequently, the Clinical Genome Resource (ClinGen) consortium focused on the development of standards for assessing genomic variation by defining evidence types for particular genes and diseases with the creation of Clinical Domain Working Groups (https://clinicalgenome.org/working-groups/clinical-domain/) and Variant Curation Expert Panels (VCEP; https://clinicalgenome.org/affiliation/).^11^ In a circular process, ClinGen’s Sequence Variant Interpretation Working Group (SVI) issues recommendations on the general application of the ACMG/AMP codes, whereas VCEPs address their gene-specific adaptations.^12^^,^^13^

The Evidence-based Network for the Interpretation of Germline Mutant Alleles (ENIGMA)^14^ was one of the first expert groups recognized by ClinGen. Recently, in the process of becoming the ENIGMA *BRCA1* and *BRCA2* VCEP, they revised and adapted their guidelines to the ACMG/AMP codes using statistical methods to calibrate evidence strength for different data types and considering recommendations of the SVI: ClinGen ENIGMA *BRCA1* and *BRCA2* Expert Panel Specifications to the ACMG/AMP Variant Interpretation Guidelines Version 1.1.0 (ENIGMA VCEP specifications).^15^^,^^16^

Several reclassification studies in different populations and for different genes, including *BRCA1* and *BRCA2*, have shown that variant reassessment based on new annotation data using the ACMG classification system can reclassify approximately 10% to 20% of VUS into the likely B (LB)/B classes (80%-90%) or the likely P (LP)/P classes (5%-10%).^17^, ^18^, ^19^, ^20^ A recent study has shown that reclassification of VUS specifically in the *BRCA1* and *BRCA2* genes using the new ENIGMA specifications results in LB/B classification of 94.8% and LP/P classification of 5.2%.^21^ The authors, however, did not specify whether the variant reclassification was triggered by the ENIGMA specifications or just by the use of the latest variant-related annotations (ie, new data). Even in the ENIGMA pilot study, which was conducted as part of the VCEP approval process, it was not possible to determine the extent to which new data were relevant for reclassification compared with the new specifications. Thus, 8 of 13 carefully selected VUS/conflicting variants from ClinVar could be reclassified as LB/B, whereby 37 LP/P and LB/B variants did not change direction and remained in their respective class (LP/P or LB/B).^15^ In this study, we compared the ratio of reclassified VUS using the new ENIGMA specifications and the standard ACMG/AMP classification system, considering the use and weighting of the specific ACMG codes. A total of 121 VUS in the *BRCA1* and *BRCA2* genes detected as part of a diagnostic test for hereditary breast and ovarian cancer were included.

For the interpretation of a genetic variant with the ENIGMA specifications, data extracted from multiple Excel tables with variant level information and many other sources of information are needed, making it difficult to access and use genomic information correctly. The University of California Santa Cruz (UCSC) Genome Browser hosts a large collection of annotations from multiple data sources and conveniently displays up-to-date data as a series of tracks aligned with the human genome sequence; at the same time, its Recommended Track Set feature facilitates the interpretation of variants in the clinic offering quick access to relevant data sets at the appropriate scale.^22^^,^^23^ To facilitate the evaluation of variants, we created a public track hub that contains ENIGMA *BRCA1*/*BRCA2* VCEP specification data. Moreover, we created a public track session (*BRCA1*/*BRCA2* ENIGMA track set) to access additional data used for the classification of sequence variants in the *BRCA1* and *BRCA2* genes following the ENIGMA *BRCA1*/*BRCA2* VCEP specifications (https://genome.ucsc.edu/s/abenet/BRCA.ENIGMA.hg19).

## Materials and Methods

### *BRCA1/BRCA2* ENIGMA track set

#### Patients and variant data

The ENIGMA track hub (https://hgdownload.soe.ucsc.edu/hubs/enigma/hub.txt) was made up of 5 new tracks containing the *BRCA1* and *BRCA2* variants curated and listed by the ENIGMA specification files from the ClinGen Criteria Specification Registry (https://cspec.genome.network/cspec/ui/svi/doc/GN092). Data (Specifications_Table9_V1.1_2023-11-22: Specifications Table9 Excel document for v1.1., Supplementary_Tables_V1.1_2023-11-22: Supplementary Tables Excel document for v1.1, Specifications_Table4_V1.1_2023-11-22: Specifications Table4 Excel document for v1.1, Specifications_V1.1_2023-11-22: Specifications document for v1.1 and Dines et al^24^) were downloaded from the ClinGen criteria specification registry: https://cspec.genome.network/cspec/ui/svi/doc/GN092. Data from supplemental tables from Parsons et al,^25^ Caputo et al,^26^ Easton et al,^27^ and Li et al^28^ were downloaded from the respective publications. Variants (in HGVS nomenclature) were mapped to the human genome hg19 and hg38 assemblies using the utility hgvsToVcf (https://github.com/imgag/ngs-bits/blob/master/doc/tools/HgvsToVcf.md). For the multifactorial likelihood analysis track NM_007294.3 and NM_000059.3 were used for mapping, as described in the original article. For the functional assays track the more recent NM_007294.4 and NM_000059.4 were used instead. These data were then reformatted from vcf to bed and converted to bigBed format. All track creation steps are documented in the track description page and are publicly available on a GitHub repository (https://github.com/ucscGenomeBrowser/kent/blob/master/src/hg/makeDb/doc/enigma.txt).

**Figure 1:**
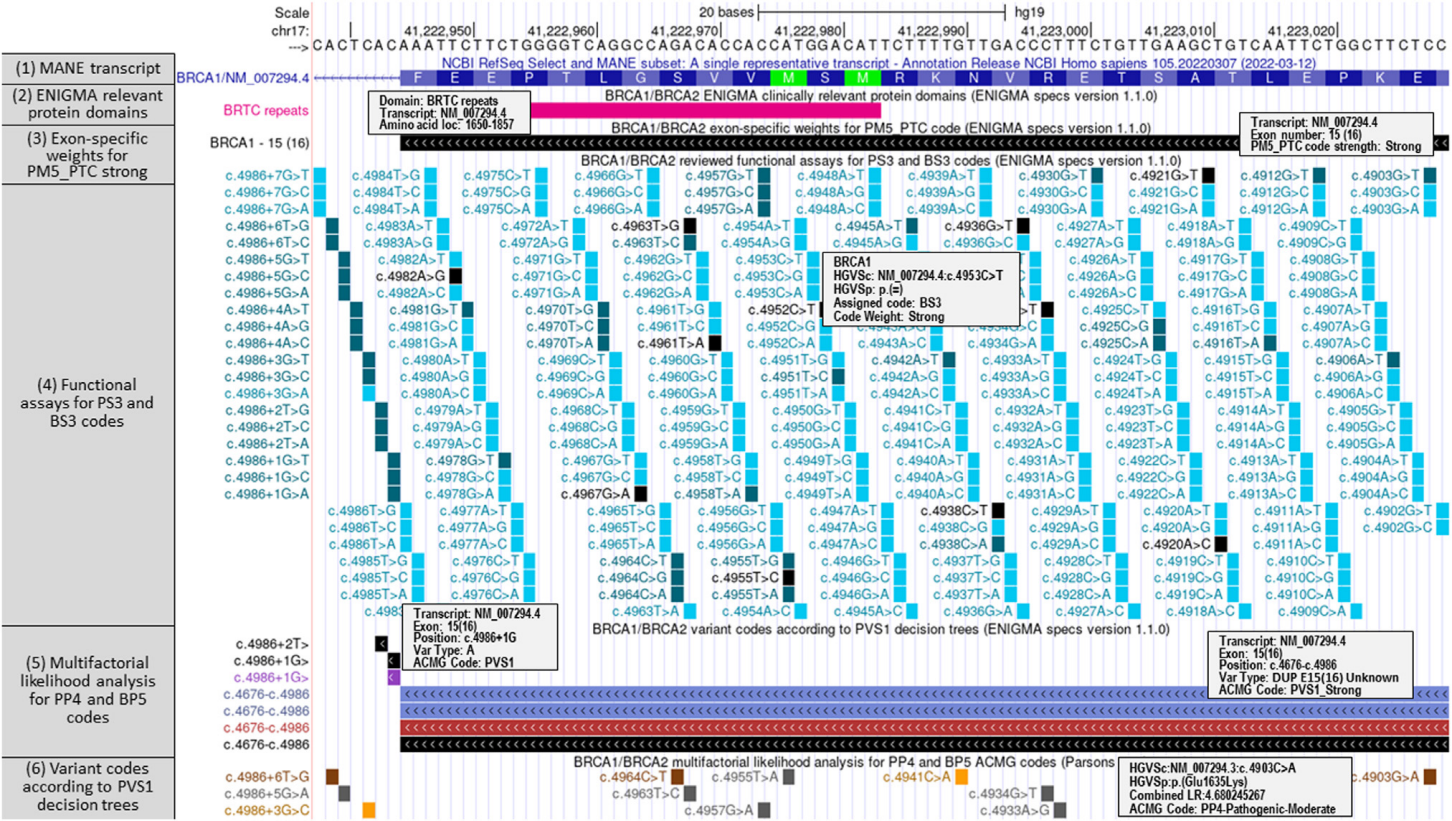
**UCSC Genome Browser Evidence-based Network for the Interpretation of Germline Mutant Alleles** **(ENIGMA) *BRCA1*/*BRCA2* track hub.** Display of the new ENIGMA tracks using ENIGMA specifications version 1.1.0 data (https://genome.ucsc.edu/s/abenet/BRCA.ENIGMA.hg19). From top to bottom tracks show the following: (1) RefSeq Select or MANE Select transcript *BRCA1*: NM_007294.4 (blue), (2) regions of *BRCA1* (and *BRCA2*) defined by ENIGMA to contain (potentially) clinically important functional domains (fuchsia items), (3) exon-specific weights for the repurposing of PM5 code (black items), (4) variants with reviewed functional assays for application of the PS3 and BS3 codes supporting or refuting a damaging effect on the gene/gene product (dark cyan: PS3, light cyan: BS3, black: no code assigned), (5) multifactorial likelihood scores for *BRCA1* (and *BRCA2*) variants to inform ENIGMA codes PP4 and BP5^16^ (brown: PP4, orange: BP5, gray: no code assigned), and (6) variants considered against the *BRCA1* (and *BRCA2*) PVS1 decision trees, including PVS1 and PM5 codes recommended for initiation, nonsense/frameshift, deletion, duplication, and splice site (donor/acceptor ±1, 2) variants (red: exon deletions, blue: exon duplications, purple: variants with supporting RNA-based functional evidence).

The ENIGMA track hub (https://hgdownload.soe.ucsc.edu/hubs/enigma/hub.txt) was made up of 5 new tracks containing the *BRCA1* and *BRCA2* variants curated and listed by the ENIGMA specification files from the ClinGen Criteria Specification Registry (https://cspec.genome.network/cspec/ui/svi/doc/GN092) (Figure 1). Data (Specifications_Table9_V1.1_2023-11-22: Specifications Table9 Excel document for v1.1., Supplementary_Tables_V1.1_2023-11-22: Supplementary Tables Excel document for v1.1, Specifications_Table4_V1.1_2023-11-22: Specifications Table4 Excel document for v1.1, Specifications_V1.1_2023-11-22: Specifications document for v1.1 and Dines et al^24^) were downloaded from the ClinGen criteria specification registry: https://cspec.genome.network/cspec/ui/svi/doc/GN092. Data from supplemental tables from Parsons et al^25^, Caputo et al,^26^ Easton et al,^27^ and Li et al^28^ were downloaded from the respective publications. Variants (in HGVS nomenclature) were mapped to the human genome hg19 and hg38 assemblies using the utility hgvsToVcf (https://github.com/imgag/ngs-bits/blob/master/doc/tools/HgvsToVcf.md). For the multifactorial likelihood analysis track NM_007294.3 and NM_000059.3 were used for mapping, as described in the original article. For the functional assays track the more recent NM_007294.4 and NM_000059.4 were used instead. These data were then reformatted from vcf to bed and converted to bigBed format. All track creation steps are documented in the track description page and are publicly available on a GitHub repository (https://github.com/ucscGenomeBrowser/kent/blob/master/src/hg/makeDb/doc/enigma.txt).

For the determination of the exon level code frame on the UCSC Genome Browser, the phase is now shown when hovering the cursor on the exons of the RefSeq genes track. The position of an exon/intron boundary within a codon is defined as the start phase and end phase: 0 between codons, 1 between the first and second base, 2 between the second and third base, and −1 for noncoding exons.

Existing tracks that provide evidence for variant interpretation according to the ACMG/AMP recommendations (ie, gene location and gene sequence, clinical variation, literature, population variation, and computational predictive data) and the new ENIGMA tracks were compiled in a browser session (https://genome.ucsc.edu/s/abenet/BRCA.ENIGMA.hg19) (Supplemental Figure 1). A comprehensive list of all tracks and associated data sets is shown in Supplemental Table 1. Each annotation track has an associated page that contains a full description of the track (display conventions, data sources, time stamp of the last data update, methods, and credits), and configuration options to fine-tune the information displayed in the track. Clicking a data item (line or box) within the track shows an associated details page. This page generally contains information specific to the item and related links to outside sites.

**Figure 2:**
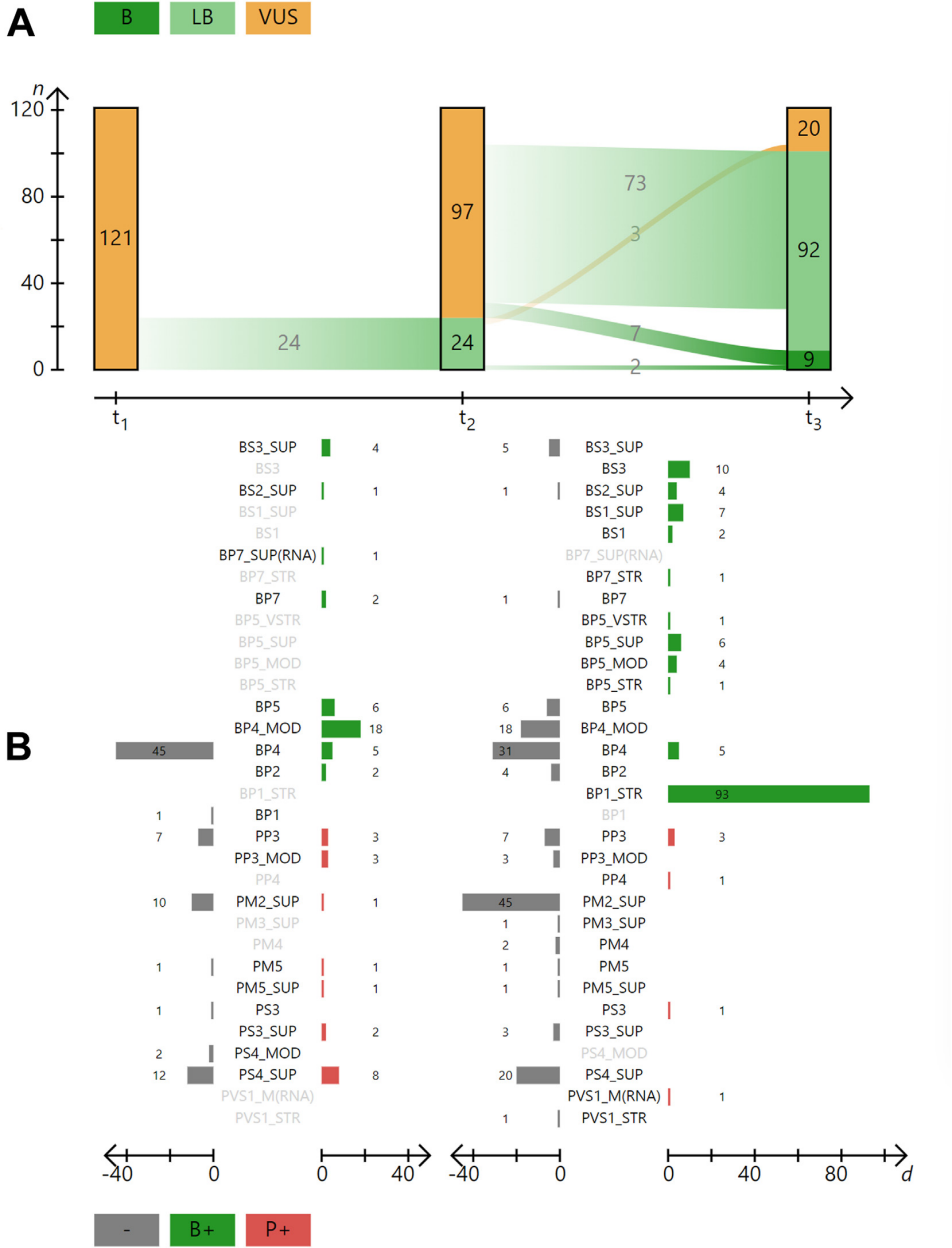
**Reclassification of VUS at 3 distinct transition points (ACMG/AMP, ACMG/AMP + SVI, and ENIGMA):** A. The top section of the figure shows the number of variants *n* as bars on the *y*-axis and their classification into (benign, likely benign, and variants of uncertain significance [VUS]) in their corresponding color (green, light green, and yellow, respectively) at 3 distinct transition points t1, t2, and t3 (*x*-axis). The transition points refer to the initial interpretation and the 2 reanalysis: American College of Medical Genetics and Genomics/Association for Molecular Pathology (ACMG/AMP) 2015 (t1) represent the baseline VUS for this study. These variants were classified as VUS between January 2018 and November 2023. Sequence Variant Interpretation Working Group (SVI) recommendations from 2015 onward were considered, depending on the time of classification, eg, PM2 used as supporting evidence strength for all variants. ACMG/AMP + SVI (t2) lists variants that have been reclassified using new annotated data as of November 2023 (eg, ClinVar, BRCA Exchange, literature, and in-house data) and using all current recommendations from the SVI as of November 2023. Evidence-based Network for the Interpretation of Germline Mutant Alleles (ENIGMA) Variant Curation Expert Panels (t3) are variants that have been reclassified based on the same new data as in t2 but using the ENIGMA specifications. The transient lines between the 3 bars, and the numbers indicate how many variants have changed from one class to another between 2 time points. B. The bottom section shows the changes in the applied ACMG/AMP codes between the transition t1/t2 and between t2/t3. On the *y*-axis are the different ACMG/AMP codes aligned. The *x*-axis depicts to the left (negative) the number of variants d for which this ACMG/AMP code was removed and to the right (positive) the number of variants for which this ACMG/AMP code was added during classification (see also Supplemental Table 3). The color distinguishes the ACMG/AMP codes that have been added and represent benign (green) and pathogenic evidence (red).

All patients met the genetic testing requirements of the German cancer guideline program.^29^ In total, 121 VUS in the genes *BRCA1* (40 VUS, NM_007294.4, OMIM 113705, HGNC:1100) and *BRCA2* (81 VUS, NM_000059.4, OMIM 600185, HGNC:1101) from 120 patients, mainly from the South German region, who underwent diagnostic next-generation-sequencing-based exome sequencing, were extracted from our internal variant database. This data set included variants classified as VUS from January 2018 to November 2023. The annotation and classification of the variants corresponded to the original ACMG/AMP classification system^12^ and the SVI recommendations available at the time of sample analysis (Figure 2A, t1; Supplemental Table 2, start point).

### Reclassification of VUS with the ACMG/AMP classification system using SVI recommendations and new data (ACMG/AMP + SVI)

The 121 variants analyzed in this study were classified as VUS using the standard ACMG/AMP classification system and SVI recommendations available at the time of interpretation (Figure 2A, t1; Supplemental Table 2, start point). The classification was performed by different variant analysts between 2018 and 2023; therefore, different SVI recommendations regarding use, weighting, and code combination rules were considered, as well as annotated data and publications available at the time of sample analysis. In addition, the MetaSVM predictor was used until December 2020 and was replaced by REVEL, resulting in differences in the use of bioinformatics predictors. These factors, which are common in laboratory practice, are the reason why VUS in T1 may lack a consistent classification.

Reclassification of VUS was conducted in a 2-step fashion. First, variants were reclassified using the standard ACMG/AMP classifications system with the latest SVI recommendations (as of November 2023) according to the latest variant-related annotations (ie, new data) (Figure 2A, t2; Supplemental Table 2; ACMG/AMP + SVI). The Clinical SNVs Recommended Track Set^23^ was used for the interpretation of variants with latest data from clinical databases (ClinVar, Leiden Open Variation Database, and Human Gene Mutation Database public version), publications (Avada and Mastermind), variant frequency data (gnomAD), predictions of pathogenicity (REVEL) and to analyze the sequence context (eg, other pathogenic amino acid exchanges at this position), and the BRCA Exchange database (Supplemental Figure 1). All reclassifications were carried out by a single experienced variant analyst to rule out inter-analyst bias (eg, multiple variant analysts could lead to inconsistent interpretations of the ACMG/AMP codes).

We used REVEL^30^ scores for the prediction of pathogenicity PP3 and BP4 (ie, computational evidence supporting a deleterious effect or no impact on the gene or gene product, respectively) and followed SVI recommendations to use the thresholds published by Pejaver et al,^31^ which allows an evidence strength beyond “supporting” in both directions (pathogenic and benign). For the use of PM2_SUP (ie, absent from controls in an outbred population [noncancer]), a maximum allele count of ≤5 in gnomAD noncancer V2.1.1 was set as threshold (eg, the most frequently described pathogenic missense variant in *BRCA1* p.(Cys61Gly) has an allele count of 6 in gnomAD noncancer v.2.1.1). For the use of PS4 (ie, the prevalence in affected individuals is significantly increased compared with controls), the proband counting described by CanVIG-UK gene-specific guidance v.1.19 was used for ≥5 described breast and ovarian cancer cases (https://www.cangene-canvaruk.org/gene-specific-recommendations).^32^

The scoring system published by Tavtigian et al^33^ was used to determine the ACMG/AMP class, which suggests a classification as likely benign from −1 point (corresponds to a supporting benign criterion). For this reason and for the sake of comparability with the ENIGMA VCEP approach, we have considered all applicable codes, even if they represent conflicting evidence (eg, PM2_SUP together with BP4). An advantage of this scoring system is that it can deal with conflicting evidence.

### Reclassification of VUS with the ENIGMA VCEP specifications (ENIGMA VCEP)

Second, all variants were reclassified using the recently published ENIGMA specifications (Expert ClinGen ENIGMA *BRCA1* and *BRCA2* Expert Panel Specifications to the ACMG/AMP Variant Interpretation Guidelines for *BRCA1* and *BRCA2* Version 1.1.0; https://cspec.genome.network/cspec/ui/svi/affiliation/50087) (Figure 2A, t3; Supplemental Table 2; ENIGMA VCEP). The specifications include (1) criteria for the determination of BS3 and PS3 based on reviewed functional assay results that support or reject damaging effect on the gene or gene product,^34^^,^^35^ (2) criteria for PM5_PTC (ie, protein termination codon variants) based on exon-specific weights, (3) a summary of codes applicable for +/−1 or 2 splice site variants considered against the *BRCA1* and *BRCA2* PVS1 decision trees, and (4) definition of clinically relevant protein domains. The calculated combined LR score from Parsons et al^25^ was used to obtain the values for PP4 and BP5, that evaluate the patient’s phenotype in relation to the specificity of a disease with a single genetic etiology or with an alternative molecular basis for the disease. SpliceAI^36^ scores were used to evaluate splicing predictions according to the specified cutoff values (ENIGMA Specifications_V1.1_2023-11-22: Figure 2A, page 19). BayesDel^37^ prediction scores were used to evaluate missense variants in clinically relevant protein domains according to the specified cutoff values for PP3 and BP4 (ENIGMA Specifications_V1.1_2023-11-22: Figure 2A, page 19). For the determination of multifactorial data regarding lack or confirmation of cosegregation (PP1 and BS4), data from the tool COOL were used.^38^ To enable better comparability with ACMG/AMP + SVI, we used the scoring system published by Tavtigian et al^33^ for the ENIGMA VCEP classification (ENIGMA VCEP; see ENIGMA Specifications_V1.1_2023-11-22: Table 3, page 14). However, a significant difference between the point-based combining rules of ENIGMA VCEP and ACMG/AMP + SVI is that a likely benign classification in the ENIGMA VCEP requires −2 points, which corresponds to at least 2 supporting benign criteria (see ENIGMA Specifications_V1.1_2023-11-22: page 14-15).

All variants were submitted to the ClinVar database (https://www.ncbi.nlm.nih.gov/clinvar/).

## Results

### The *BRCA1/BRCA2* ENIGMA track set

The *BRCA1*/*BRCA2* ENIGMA track set contains the data required for the correct assignment of the ACMG/AMP codes to *BRCA1* and *BRCA2* variants according to the modification of the ACMG/AMP code usage recommended by ENIGMA (Expert ClinGen ENIGMA *BRCA1* and *BRCA2* Expert Panel Specifications to the ACMG/AMP Variant Interpretation Guidelines for *BRCA1* and *BRCA2* Version 1.1.0). This group is composed of a total of 14 tracks, 5 of which have been created specifically to address this topic (https://genome.ucsc.edu/s/abenet/BRCA.ENIGMA.hg19) (Supplemental Table 1, Supplemental Figure 1). The 5 new tracks display all variants included in the ENIGMA specifications sheets (https://cspec.genome.network/cspec/ui/svi/doc/GN092). Each variant is associated with a description page showing all the information from the ENIGMA tables, and hovering over specific elements displays the ACMG/AMP code and the strength applied to the variant, among other details (Figure 1).

The track set can be activated in the GRCh37/hg19 and GRCh38/hg38 assemblies, from the main menu under Genome Browser, by clicking on Recommended Track Sets in the drop-down list or under My Data, by clicking on Track Hubs and search for “ENIGMA BRCA1/BRCA2 specs 1.1.0.”.

### Reclassification of variants using the ACMG/AMP classification system with SVI recommendations (ACMG/AMP + SVI)

Of 121 VUS, 20% (*n* = 24) were reclassified as LB based on current annotation data and using the current recommendations of the SVI and the standard ACMG/AMP classification system (ACMG/AMP + SVI), whereas 80% (*n* = 97) remained as a VUS (Figure 2A; t2). No variants were reclassified as LP/P. It should be noted that the classification of variants in the baseline (t1) was performed at different times between 2018 and 2023 and therefore used different iterations of the SVI guidelines and annotation data available at the time of assessment. During this time (ie, 2018 to 2023), classified VUS were not reclassified as new guidelines, or new data emerged; therefore, some VUS statuses may be inconsistent. Still, our results are in line with the data from other studies.^17^, ^18^, ^19^, ^20^^,^^39^

In ACMG/AMP + SVI, it is noticeable that the codes for bioinformatic prediction (BP4 and PP3) were used less frequently in their original (supporting) strength (BP4: −45 and PP3: −7). Instead, the evidence strength was frequently increased to “moderate” for both codes (BP4_MOD: +18 and PP3_MOD: +3), which can be attributed to the extension of the evidence strength depending on the REVEL score recommended by Pejaver et al.^31^

Variant reannotation and comparison with ClinVar, BRCA Exchange, and the literature led to the identification of further reported individuals with BC, allowing the use of the code PS4_SUP (proband counting) for 8 variants. However, the stricter interpretation in the use of PS4 as suggested by the SVI (ie, it must be clear from the source that the individuals were affected, and the variant must not be listed too frequently in population databases) resulted in PS4_SUP being removed for 12 variants. The less frequent use of the code PM2_SUP (−10) is attributable to more recent data from the gnomAD noncancer data sets (Figure 2B; t2 and Supplemental Table 3)

### Reclassification of variants using the ENIGMA specifications (ENIGMA VCEP)

Using the ENIGMA specifications (ENIGMA VCEP) after ACMG/AMP + SVI reclassification with new variant data (ACMG/AMP + SVI), 83.5% (*n* = 101) of VUS were reclassified as B (*n* = 9) or LB (*n* = 92) (Figure 2A; t3). Of note, 3 VUS at t1 that could be reclassified to LB using ACMG/AMP + SVI (t2), returned to a VUS using ENIGMA VCEP (t3). No variant was reclassified as LP/P. We observe a similar reclassification rate toward LB/B for *BRCA1* (85%, *n* = 40) and *BRCA2* (83%, *n* = 67).

The large difference in the rate of down-classifications with ENIGMA VCEP compared with ACMG/AMP + SVI is mainly explained using the code BP1_STR, which could be applied to 93 variants (Figure 2B, t3; Supplemental Table 3). In the absence of other evidence, the use of BP1_STR (−4 points) results in a classification as LB. Even if these variants fulfill the rarity criteria PM2_SUP (+1 point), it still results in a classification as LB, because −3 points correspond to class 2 (see ENIGMA Specifications_V1.1_2023-11-22: page 14-15).

The BP5 code could be applied to 12 variants (1 × BP5_VSTR, 1 × BP5_STR, 4 × BP5_MOD, and 6 × BP5_SUP) as the ENIGMA specifications consider using the multifactor likelihood scores from Parsons et al,^25^ Caputo et al,^26^ Easton et al,^27^ and Li et al.^28^ In addition, the BS3 code could be used for 10 variants given the functional data available for these variants (see ENIGMA Specifications_Table9_V1.1_2023-11-22: Specifications Table9 Excel document for v1.1) (Supplemental Table 3).

Another aspect of the ENIGMA specifications is the usage of the code BP4 (missense predictions using BayesDel). BP4 can only be applied to missense variants in the domains defined by ENIGMA as potentially clinically important (see ENIGMA Specifications_V1.1_2023-11-22: Figure 2A, page 19) and is restricted to “supporting” strength. This specification caused a more limited usage of this code, which led to an overall reduced application of BP4/BP4_MOD (BP4: −31 and BP4_MOD: −18) compared with ACMG/AMP + SVI, in which BP4 could be applied without any restriction regarding its location in the gene (Figure 2B, Supplemental Tables 2 and 3). It is noticeable that BP4 for missense variants could be applied only for *BRCA2* variants (*n* = 10), all of which are in the C-terminal DNA-binding domain.

The stricter definition of the PM2_SUP criterion in the ENIGMA specifications, which only applies to variants absent from gnomAD V2.1.1 or 3.1.2 noncancer data sets, led to a reduction in the use of this code (PM2_SUP: −45) (Figure 2B, Supplemental Tables 2 and 3). Furthermore, the lack of published data on case-control studies together with the omission of the proband-counting option in ENIGMA VCEP resulted in PS4_SUP (−20) not being used (Figure 2B, Supplemental Tables 2 and 3).

## Discussion

A VUS can be reclassified using 2 fundamentally different and complementary approaches: (1) new variant annotation data (population allele frequencies, functional studies, case-control data, segregation analysis, pathogenicity prediction, etc) can be gathered to reevaluate a variant and ultimately reclassify it using the same classification system, taking recommendations from the SVI into account; (2) for some genes/diseases, VCEPs have recently published gene-specific adaptations of the standard ACMG/AMP codes. They not only define whether and to what extent the codes are used (code usage and evidence strength), but they may also specify the use of data sets and significant thresholds (eg, functional studies), the application of multifactorial likelihood values (currently only used by the InSiGHT Hereditary Colorectal Cancer/Polyposis VCEP and the ENIGMA VCEP to our knowledge), and the cutoffs for frequency data. This latter approach has proven to be very effective and has led to a better classification of variants processed by the VCEPs.^15^

Similar to Innella et al,^21^ our results show a profound reclassification rate of *BRCA1* and *BRCA2* variants in a clinical data set using the ENIGMA VCEP specifications. Moreover, we demonstrate and detail the gain in information that can be achieved by applying VCEP specifications over the ACMG classification system using new annotated data. The article by Innella et al^21^ did not identify the individual factors that led to a reclassification of the variants. Because their data set spanned more than a decade, a reclassification could have been triggered only by new annotated data (eg, publication of functional studies, gnomAD data, and patients with breast and ovarian cancer) rather than by VCEP specifications. Our data show that new data reclassify a few variants compared with the use of ENIGMA VCEP specifications. This is of relevance because most clinical diagnostic laboratories still do not use VCEP expert specifications in general, nor for *BRCA1* and *BRCA2*, as documented in the ClinVar submissions “Review status (Assertion criteria).”

We show that using ENIGMA specifications dramatically reduces the number of VUS (83.5% vs 20% with the same new data but standard ACMG/AMP + SVI). All of these could be reclassified in the 2 benign classes (LB/B), clearly demonstrating the efficacy of ENIGMA VCEP over new-data ACMG/AMP + SVI, which is presumably a consequence of working in a rare disease environment. Of the current 2124 *BRCA1* and 2953 *BRCA2* VUS in ClinVar, a total of 1280 *BRCA1* (60.3%) and 2282 *BRCA2* (77.3%) VUS are unique submissions (as of June 2024). The high rate of VUS that could be reclassified to LB/B based on the ENIGMA VCEP definition of code BP1 (ie, missense variant in a gene for which disease-causing variants are primarily truncating) may appear to be a rare exception only relevant for *BRCA1* and *BRCA2*. However, we would like to emphasize that a similar situation applies to all genes in which missense variants are generally not an established pathomechanism or can cause disease in only certain functional domains of the protein. The recently published expert specifications for *APC* (https://cspec.genome.network/cspec/ui/svi/doc/GN089) and *PALB2* (https://cspec.genome.network/cspec/ui/svi/doc/GN077) serve here as an example. For both genes, the BP1 code can be applied; therefore, we also expect a considerable reduction of the VUS toward LB/B just by virtue of the definition of this code. These examples should draw attention to the fact that the use of VCEP specifications is generally preferable to the standard ACMG classification system.

The reverse case (eg, a VUS at t1 changes to a LB in ACMG/AMP + SVI (t2) but returns to a VUS in ENIGMA VCEP (t3)) applies for only 3 variants. In case 1 (*BRCA2* NM_000059.4:c.8668C>A, p.(Leu2890Ile); Supplemental Table 2, patient 109), a more conservative interpretation of ENIGMA regarding the use of code BP5 in cooccurrence with another pathogenic variant (ie, *BRCA1* and *BRCA2* pathogenic variants in 1 patient) causes a different classification compared with ACMG/AMP + SVI. Of note, when using the Bayesian point-based system, both approaches result in −1 point, which qualifies as a LB in ACMG/AMP + SVI but as a VUS in ENIGMA VCEP because of the different thresholds. We are aware that laboratories may use these codes or their weighting differently and emphasize that this is also an advantage of Expert Panel specifications, which define uniform and unambiguous use, weighting, and combination of codes. Because it is not uncommon to observe a pathogenic variant in *BRCA1* and *BRCA2* in the same patient, the use of BP5 in ACMG/AMP + SVI might be questionable for such scenarios.^40^^,^^41^ In case 2 (*BRCA2* NM_007294.4:c.830A>G, p.(Asn277Ser); Supplemental Table 2, patient 106), the use of REVEL in ACMG/AMP + SVI allowed the application of BP4 in moderate evidence strength, whereas for ENIGMA VCEP SpliceAI was conspicuous and suggests application of PP3 (for splicing). Evaluation with BayesDel is not permitted, because this variant is not located in a domain defined as potentially clinically important by ENIGMA. Case 3 (*BRCA1* NC_000017.11, NM_007294.4:c.4096+4T>C; Supplemental Table 2, patient 50) concerns an intronic variant, which according to ENIGMA, is only assessed as BP4 with a negative SpliceAI score, whereas ACMG/AMP + SVI allows use of BP4 and BP7. In no case was a VUS reclassified to a LP/P, neither with ENIGMA VCEP nor with ACMG/AMP + SVI.

In this study, we have focused on VUS but would like to emphasize that LB/B and LP/P variants should also be reevaluated periodically if the variants have not been conclusively assessed by a VCEP. Regular retrospective interpretation of variants considering new data annotations and new guidelines is an important burden in daily practice. Yet, in ClinVar, a monthly median of 1.247 changes in variant classification with potential clinical significance were recorded between 2017 and 2019, 89% validated by experts.^42^ We therefore recommend that laboratories implement a follow-up policy for sequence variant reclassification. To this end, we now offer patients presenting to our laboratory for disease follow-up reclassification of sequence variants with the corresponding VCEP specifications.

With the newly developed *BRCA1*/*BRCA2* ENIGMA track set, we provide the community with a valuable tool for variant classification in the *BRCA1* and *BRCA2* genes. Because the process of biocuration is slow and laborious, many VUS that could be reclassified as LB/B will remain in ClinVar. With the *BRCA1*/*BRCA2* ENIGMA track set, we want to make it convenient for variant analysts to obtain the relevant data for this process. Considering all the information needed to interpret every single variant, the UCSC Genome Browser interface with *BRCA1* and *BRCA2* ENIGMA data points aligned to the human genome assembly significantly simplifies the visualization of annotations at variant and exon level and facilitates the correct use of the ENIGMA specifications. The schematic presentation of the relevant data (whether the variant is contained in one of the ENIGMA sources, or it is in a potentially clinically relevant domain, multifactorial data are available, or functional data are known, etc.) considerably speeds up the process of biocuration and enhances the accuracy of genomic variant interpretation without additional costs. Before using these data, users should verify that the specification version numbers in the tracks match the latest version in ClinGen’s specification registry.

The possibility of combining different data tracks depending on the user’s needs and saving them in public sessions makes the UCSC Genome Browser a powerful tool to accommodate new emerging gene- or disease-specific VCEP guidelines. In general, the genome tracks are primarily designed to provide information for the study of inherited disorders (primarily Mendelian) and should be used with caution. These are only research tools and in no way should be used to inform medical decisions.

In conclusion, reclassification using the ENIGMA VCEP specifications leads to a dramatic reduction of VUS compared with reclassification using the standard ACMG/AMP classification system. As a result, we were able to create inconspicuous reevaluation reports for 101 patients, conclude their diagnosis and contribute to their relief. We encourage laboratories to not only use the ENIGMA VCEP specifications for the classification of variants in the *BRCA1* and *BRCA2* genes but to use all published VCEP specifications (https://www.clinicalgenome.org/working-groups/clinical-domain/).

## Data Availability

All variants were submitted to ClinVar. Full patient data are not available publicly to respect participant privacy and consent. Anonymized data not published within this article will be made available by request from any qualified investigator.

## Conflict of Interest

Luis R. Nassar and Maximilian Haeussler receive royalties from the sale of University of California Santa Cruz Genome Browser source code, LiftOver, GBiB, and GBiC licenses to commercial entities.
