# Supplementary material for: Reclassification of VUS in BRCA1 and BRCA2 using the new BRCA1/BRCA2 ENIGMA track set demonstrates the superiority of ClinGen ENIGMA Expert Panel specifications over the standard ACMG/AMP classification system
Source: Genet Med Open. 2025 Jan 7;3:101961. doi: 10.1016/j.gimo.2024.101961 (PMC11869971; doi:10.1016/j.gimo.2024.101961)
Supplement: Supplementary Figure [file mmc2.pdf]

Supplementary Figure 1: UCSC Genome Browser *BRCA1*/*BRCA2* ENIGMA track set combines recommended Clinical SNVs tracks and the ENIGMA *BRCA1*/*BRCA2* track hub. From top to bottom tracks show: (1) RefSeq Select or MANE Select transcript *BRCA1*: NM_007294.4, (2) ENIGMA tracks using ENIGMA specifications version 1.1.0 data, (3) ClinVar short variants (<50bp) and submitted interpretations, (4) Human Gene Mutation Database public variants, (5) Leiden Open Variation Database short variants (<50 bp), (5) BRCA exchange variants, (6) GnomAD non-cancer variants from exomes version 2 and GnomAD genomes version 2, (7) BayesDel missense prediction scores, (8) AVADA variants and (9) Genomenon Master Mind variants extracted from full-length publications.

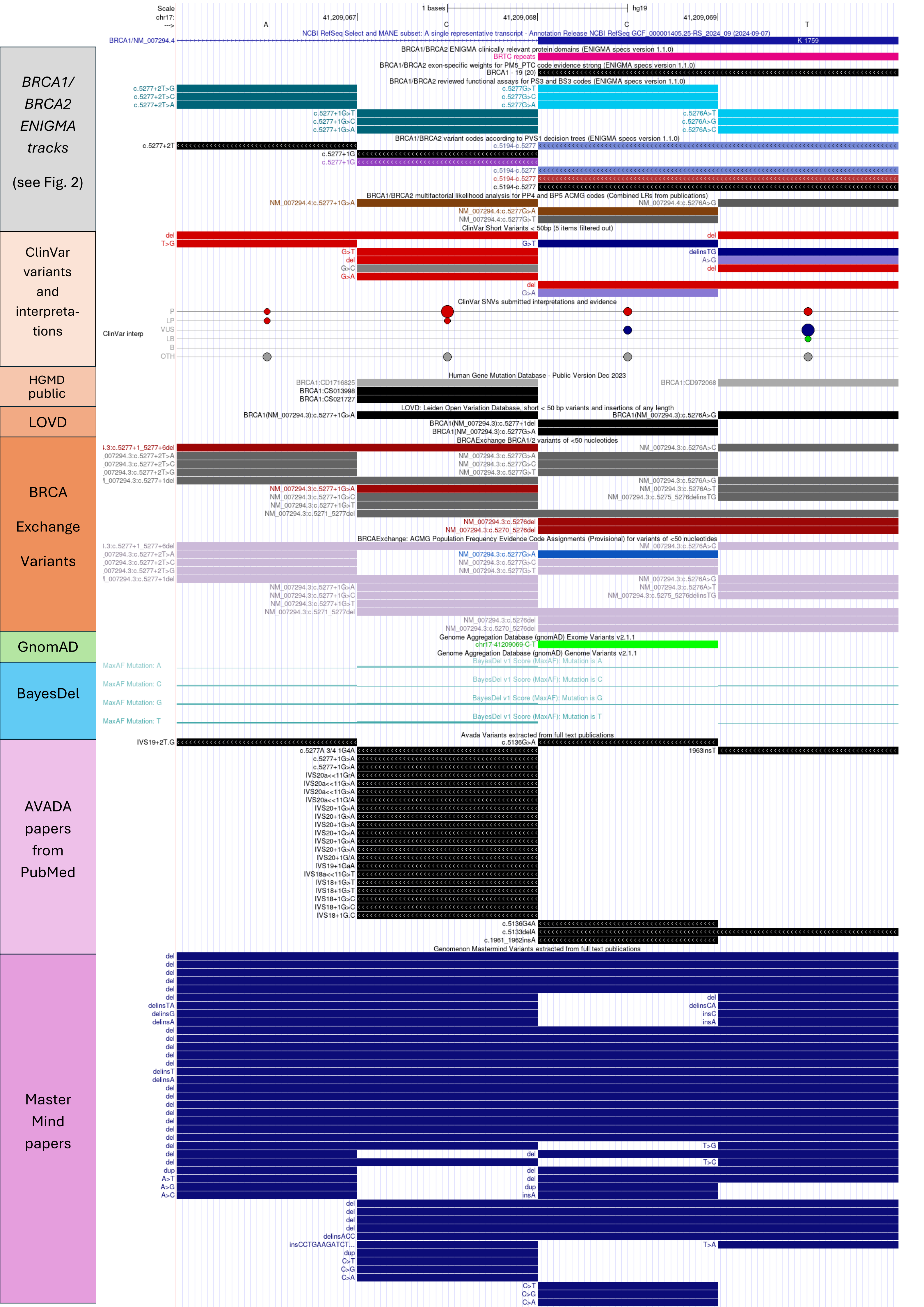

Scale
chr17:
1 bases
hg19
41,209,067
41,209,068
41,209,069
A
C
C
T
NCBI RefSeq Select and MANE subset: A single representative transcript - Annotation Release NCBI RefSeq GCF_000001405.25-RS_2024_09 (2024-09-07)
BRCA1/NM_007294.4
K 1759
BRCA1/BRCA2 ENIGMA tracks
(see Fig. 2)
BRCA1/BRCA2 ENIGMA clinically relevant protein domains (ENIGMA specs version 1.1.0)
BRTC repeats
BRCA1/BRCA2 exon-specific weights for PM5_PTC code evidence strong (ENIGMA specs version 1.1.0)
BRCA1 - 19 (20)
BRCA1/BRCA2 reviewed functional assays for PS3 and BS3 codes (ENIGMA specs version 1.1.0)
BRCA1/BRCA2 variant codes according to PVS1 decision trees (ENIGMA specs version 1.1.0)
BRCA1/BRCA2 multifactorial likelihood analysis for PP4 and BP5 ACMG codes (Combined LRs from publications)
ClinVar variants and interpreta-tions
ClinVar Short Variants < 50bp (5 items filtered out)
ClinVar SNVs submitted interpretations and evidence
ClinVar interp
P
LP
VUS
LB
B
OTH
HGMD public
Human Gene Mutation Database - Public Version Dec 2023
LOVD
LOVD: Leiden Open Variation Database, short < 50 bp variants and insertions of any length
BRCA Exchange Variants
BRCAExchange BRCA1/2 variants of <50 nucleotides
BRCAExchange: ACMG Population Frequency Evidence Code Assignments (Provisional) for variants of <50 nucleotides
GnomAD
Genome Aggregation Database (gnomAD) Exome Variants v2.1.1
chr17-41209069-C-T
Genome Aggregation Database (gnomAD) Genome Variants v2.1.1
BayesDel
MaxAF Mutation: A
MaxAF Mutation: C
MaxAF Mutation: G
MaxAF Mutation: T
BayesDel v1 Score (MaxAF): Mutation is A
BayesDel v1 Score (MaxAF): Mutation is C
BayesDel v1 Score (MaxAF): Mutation is G
BayesDel v1 Score (MaxAF): Mutation is T
AVADA papers from PubMed
Avada Variants extracted from full text publications
Master Mind papers
Genomenon Mastermind Variants extracted from full text publications
